# Implementation research for public sector mental health care scale-up (SMART-DAPPER): a sequential multiple, assignment randomized trial (SMART) of non-specialist-delivered psychotherapy and/or medication for major depressive disorder and posttraumatic stress disorder (DAPPER) integrated with outpatient care clinics at a county hospital in Kenya

**DOI:** 10.1186/s12888-019-2395-x

**Published:** 2019-12-28

**Authors:** Rachel Levy, Muthoni Mathai, Purba Chatterjee, Linnet Ongeri, Simon Njuguna, Dickens Onyango, Dickens Akena, Grace Rota, Ammon Otieno, Thomas C. Neylan, Hafsa Lukwata, James G. Kahn, Craig R. Cohen, David Bukusi, Gregory A. Aarons, Rachel Burger, Kelly Blum, Inbal Nahum-Shani, Charles E. McCulloch, Susan M. Meffert

**Affiliations:** 1 Medical School, University of California, San Francisco, CA USA; 2 Department of Psychiatry, University of Nairobi, Nairobi, Kenya; 3 Department of Obstetrics, Gynecology and Reproductive Sciences, University of California, San Francisco, CA USA; 4 Kenya Medical Research Institute (KEMRI), Nairobi, Kenya; 5 Director of Mental Health, Kenyan Ministry of Health, Nairobi, Kenya; 6 Kisumu County, Ministry of Health, Kisumu, Kenya; 7 Department of Psychiatry, Makerere University, Kampala, Uganda; 8 University of Nairobi, Nairobi, Kenya; 9 Departments of Psychiatry and Neurology, University of California, San Francisco, CA USA; 10 Division of Mental Health and Control of Substance Abuse, Ministry of Health –, Kampala, Uganda; 11 Department of Epidemiology and Biostatistics, Institute for Health Policy Studies, University of California, San Francisco, CA USA; 12 Department of Obstetrics, Gynecology and Reproductive Sciences, University of California, UC Global Health Institute, San Francisco, CA USA; 13 Department of Psychiatry, Kenyatta National Hospital, University of Nairobi, Nairobi, Kenya; 14 Department of Psychiatry, University of California, San Diego, CA USA; 15 Department of Psychiatry, University of California, San Francisco, CA USA; 16 Institute for Social Research, University of Michigan, Ann Arbor, Michigan USA

**Keywords:** Depression, Post-traumatic stress disorder (PTSD), Trauma, Interpersonal psychotherapy (IPT), SSRI, Fluoxetine, Primary care, Implementation science, Sequential, Multiple assignment randomized trial, Sub-Saharan Africa

## Abstract

**Background:**

Mental disorders are a leading cause of global disability, driven primarily by depression and anxiety. Most of the disease burden is in Low and Middle Income Countries (LMICs), where 75% of adults with mental disorders have no service access. Our research team has worked in western Kenya for nearly ten years. Primary care populations in Kenya have high prevalence of Major Depressive Disorder (MDD) and Posttraumatic Stress Disorder (PTSD). To address these treatment needs with a sustainable, scalable mental health care strategy, we are partnering with local and national mental health stakeholders in Kenya and Uganda to identify 1) evidence-based strategies for first-line and second-line treatment delivered by non-specialists integrated with primary care, 2) investigate presumed mediators of treatment outcome and 3) determine patient-level moderators of treatment effect to inform personalized, resource-efficient, non-specialist treatments and sequencing, with costing analyses. Our implementation approach is guided by the Exploration, Preparation, Implementation, Sustainment (EPIS) framework.

**Methods/design:**

We will use a Sequential, Multiple Assignment Randomized Trial (SMART) to randomize 2710 patients from the outpatient clinics at Kisumu County Hospital (KCH) who have MDD, PTSD or both to either 12 weekly sessions of non-specialist-delivered Interpersonal Psychotherapy (IPT) or to 6 months of fluoxetine prescribed by a nurse or clinical officer. Participants who are not in remission at the conclusion of treatment will be re-randomized to receive the other treatment (IPT receives fluoxetine and vice versa) or to combination treatment (IPT and fluoxetine). The SMART-DAPPER Implementation Resource Team, (IRT) will drive the application of the EPIS model and adaptations during the course of the study to optimize the relevance of the data for generalizability and scale –up.

**Discussion:**

The results of this research will be significant in three ways: 1) they will determine the effectiveness of non-specialist delivered first- and second-line treatment for MDD and/or PTSD, 2) they will investigate key mechanisms of action for each treatment and 3) they will produce tailored adaptive treatment strategies essential for optimal sequencing of treatment for MDD and/or PTSD in low resource settings with associated cost information – a critical gap for addressing a leading global cause of disability.

**Trial registration:**

ClinicalTrials.gov NCT03466346, registered March 15, 2018.

## Background

Mental disorders are a leading cause of disability globally [1], largely driven by depression and anxiety [2, 3]. Most of the disease burden is in Low and Middle Income Countries (LMICs), where 75% of adults with mental disorders have no service access [4]. Despite nearly 15 years of efficacy research showing that local non-specialists can provide evidence-based care for depression and anxiety in LMICs [5–7], few studies have advanced to the critical next step and morbidity from mental disorders continues to escalate [8–11]. It is vital that global mental health treatment researchers now focus on implementation science to inform scale-up of evidence-based care to lower mental health burden. As emphasized by a recent World Health Organization (WHO) initiative [12], integration of mental health treatment into existing systems of care is critical to achieving public health impact.

With high prevalence of Major Depressive (MDD) (26% [13]) and Posttraumatic Stress Disorder (PTSD) (35%( [14)]) in primary care populations, treatment for depression and PTSD are leading concerns for Kenyan mental health policy makers. Implementation knowledge gaps thwart efforts to scale up care for depression and trauma-related disorders. Kenyan healthcare providers and policy-makers launched a government-funded initiative to scale-up treatment for mental disorders in primary healthcare [15]. Yet, they lack an evidence base to guide programs for two essential treatments –psychotherapy and second generation antidepressants [16]— without which Kenyan care scale-up will fall short of its potential [17, 18]. The study described here responds to this need.

Our research team has worked in Kenya for nearly 10 years with a UCSF-Kenya Medical Research Institute (KEMRI) collaboration (Family AIDS Care and Education Services [FACES]) that supports integrated HIV services at over 70 primary care facilities in Kisumu County. In collaboration with FACES, we conducted a randomized, controlled trial in Kisumu County of Interpersonal Psychotherapy (IPT) delivered by non-specialists for HIV-positive patients with MDD and PTSD [19]. In our study, IPT achieved full remission of MDD or PTSD in many cases—higher than in U.S. effectiveness studies with physician-delivered depression care [20]..

Implementation research on depression and trauma-disorder treatment within existing LMIC health care systems must consider not only individual treatment benefits in culturally distinct populations, but also barriers affecting access to care, healthcare system capacities, and budget. Hence, we are partnering with local, national and regional mental health stakeholders to evaluate: 1) non-specialist delivery of evidence-based depression and PTSD treatment integrated within existing healthcare centers in regards to clinical effectiveness and implementation parameters; including 2) costs and cost-benefit ratios. Our implementation approach is guided by the Exploration, Preparation, Implementation, Sustainment (EPIS) framework. EPIS describes the aforementioned phases of implementation as well as key dimensions to be considered during the implementation process [21, 22].. In addition to the process and phases of implementation, EPIS identifies the importance of outer system context, inner organizational context, “bridging factors” that link outer and inner context, and characteristics of the treatment and its fit at system, organization, provider, and patient levels. These implementation factors are crucial to implementation, sustainability and scale up in this setting. The SMART-DAPPER Implementation Resource Team, (IRT) and its associated Dynamic Adaptation Process (DAP) will drive the application of the EPIS model and allowable adaptations during the course of the study to optimize the relevance of the data for generalizability and scalability.

Given the goal of identifying a sustainable model for “real world” non-specialist treatment to reduce population-level disability caused by depression and trauma-related disorders, we will address common clinical dilemmas, such as what treatment to start with and how to modify it [20]. In particular, we will identify 1) evidence-based strategies for first-line and second-line (non-remitter) treatment delivered by non-specialists, and 2) patient-level moderators of treatment outcome to inform personalized, non-specialist tailored treatment.

With investigators from the University of California, San Francisco (UCSF), the University of Nairobi (UoN), The Kenya Medical Research Institute (KEMRI), the University of California San Diego (UCSD), Makerere University and the University of Michigan, as well as the National and Kisumu County Ministry of Health and the Ugandan Ministry of Health (mental health lead), SMART-DAPPER is collaborating with the FACES team providing services to Kisumu County Hospital (KCH) primary care outpatient clinic (~ 10,000 patients/month) to recruit 2710 adult primary care patients with MDD and/or PTSD (irrespective of HIV status) to a Sequential, Multiple Assignment Randomized Trial (SMART). Critically, KCH has the capability and commitment to sustain the intervention. Local non-specialists will be trained in mental health care for the SMART and hired through the Kisumu County Health Department to work at KCH, to further encouraged sustained care after the conclusion of the study.

In line with the implementation and scale up goals of this study, we will use the established, evidence-based standard of care for IPT (12 weekly sessions [3 months]) and fluoxetine (6 months). Participants will be randomized to one of two first-line treatment options, either 1) three months of interpersonal psychotherapy (IPT) or 2) six months of fluoxetine. Then, non-remitters at the conclusion of treatment will be re-randomized to one of two second line treatment options, either 1) switch to the alternative treatment (“switch”) (e.g., IPT to fluoxetine), or 2) add the alternative treatment (“combination”) (e.g., addition of IPT to fluoxetine). Research with care delivered in high income countries suggests that antidepressants and psychotherapy have equivalent short-term efficacy and that psychotherapy yields superior long-term relapse prevention [23–28]. We will also test key moderators of treatment effect (for example, distance from facility, severity of baseline symptoms). Results of moderator analyses using a method called “Q learning” will produce tailored first and second-line non-specialist treatment algorithms.

### Study treatments

#### Interpersonal psychotherapy (IPT)

IPT was developed in the 1980s by Gerald Klerman and Myrna Weissman to address interpersonal issues in depression [29]. IPT is now evidence-based treatment for depression and PTSD [30–38]. IPT improves symptoms by addressing problems in social relationships. IPT is traditionally delivered as weekly one-hour sessions over 12 weeks, focused on one of the following interpersonal problem areas: role conflict, role transition or interpersonal loss (grief). IPT may have two advantages for treating primary care populations in Kenya. First, IPT improves depression and trauma symptoms by addressing problems in social relationships. Given the cultural emphasis placed on social bonds across Kenya and regionally, IPT is compatible with local values, making it a scalable treatment. Second, IPT has the advantage of simultaneously treating MDD and PTSD. This is consistent with implementation frameworks that identify need to consider that fit and potential adaptations of clinical interventions to address the culture and characteristics of communities, organizations, providers, and patients [21, 22].

#### Fluoxetine

Fluoxetine treatment is associated with decreased symptoms of MDD [39, 40]. Despite the interim development of many other Selective Serotonin Reuptake Inhibitors (SSRIs), none have shown greater clinical efficacy for depression than fluoxetine, and it remains a first line treatment for depression [41]. First shown to be efficacious treatment for PTSD in 1999 [42], fluoxetine is also a first line treatment for PTSD [43]. Fluoxetine is on the Kenyan essential drugs list and is commonly used in public sector healthcare.

#### Non-specialist delivery of IPT and antidepressants in East Africa

Our research team has shown that IPT and antidepressants are acceptable, feasible and have strong efficacy when delivered by local non-specialists to regional HIV-affected populations using efficacy and pilot effectiveness-implementation hybrid designs [19, 44–49].

#### MDD, PTSD or both

MDD and PTSD are frequently co-morbid—approximately 50% of those with PTSD also have MDD [50] and 30–40% of those with MDD also have PTSD [51, 52]. Given our goal of investigating sustainable and scale-able interventions for use in “real-world” practice settings, we have intentionally selected permissive study eligibility criteria that will allow us to include participants with MDD, PTSD and both – the combinations that providers are likely to encounter in practice.

#### Rationale for SMART design

As mentioned, the purpose of this study is to identify evidence-based strategies for first-line and second-line (non-remitter) treatment delivered by non-specialists, including optimal choice of treatment sequences. SMART designs are ideal for this goal, as they were developed specifically to inform the construction of adaptive treatment strategies (ATS) [53–55]. SMARTs enable the efficient use of a given sample to address multiple scientific questions concerning ATS, unlike standard designs that require many more treatment arms (and hence many more participants) [56]..

The proposed SMART (Table 2) examines 1) first line treatment with IPT or fluoxetine, and 2) second line tactics for non-remitters—specifically, treatment “switch” (from IPT to fluoxetine or vice versa) or treatment “combination” (addition of IPT to fluoxetine or vice versa). We investigate first line treatments in regard to both post-treatment remission (see primary outcomes, below) and long-term relapse prevention. Research with specialists in High Income Countries (HICs) suggests that psychotherapy may be advantaged over antidepressant medication for relapse prevention [28, 57, 58] with ongoing controversy [59]. Given the importance of relapse prevention for efficient non-specialist treatment in low resource settings, the proposed study extends over 30 months with at least 24 months of follow-up. Understanding the relapse risks for treatments that do or do not include psychotherapy (IPT) is critical for reducing population-level mental disorder burden. Our second line tactics for non-remitters (i.e., those who continue to meet criteria for MDD, PTSD or both after initial treatment) are “switching” or “combining” treatment. “Switching” versus “combining” is a common dilemma for clinicians who must decide how to revise treatment to achieve goals. Despite its practical importance, the relative merits of “switching” versus “combining” antidepressants and psychotherapy remain underexplored globally [26, 60] and to our knowledge, have not been evaluated in the setting of non-specialist delivery in LMICs.

### Research objectives and hypotheses

We hypothesize:1) Although we expect that initial randomization to IPT or fluoxetine will have similar efficacy for post-treatment remission we will test for superiority of either intervention; 2) Participants who remit with first line IPT will have fewer relapses in follow-up assessments than those who remit with first line fluoxetine; 3) For initial treatment with IPT, change in social support will mediate the relationship between treatment and remission—improved social support will be associated with remission; 4) For initial treatment with fluoxetine, change in emotional reactivity will mediate the relationship between treatment and remission—decreased emotional reactivity will be associated with remission; 5) Time or cost for transport between participants’ residences and KCH will moderate the relationship between treatment and remission—higher time/cost will decrease the effect of initial treatment with IPT relative to fluoxetine (more KCH visits required for IPT versus fluoxetine treatment); 6) Severity of MDD and/or PTSD symptoms among non-remitters will moderate the relationship between subsequent treatment and remission, such that higher severity will decrease the effect of treatment on remission for treatment switch relative to treatment combination and; 7) Pooled cost-benefit ratios will show that depression and/or PTSD treatment leads to net economic gains.

The results of the proposed research will be significant in at least two ways: 1) they will determine the effectiveness of non-specialist delivered first- and second-line treatment for MDD and PTSD in LMICs and; 2) they will inform the optimal sequencing and adaptation of treatment for MDD and PTSD in low resource settings – a critical gap for addressing a leading global cause of disability.

## Methods/design

### Trial design

Sequential, Multiple Assignment Randomized Trial (SMART).

### Ethics

The trial has been approved by the UCSF Institutional Review Board (IRB) and the Kenyatta National Hospital-University of Nairobi Ethics and Research Committee. It is registered on ClinicalTrials.gov (NCT03466346). The trial will be conducted in accordance with Human Subjects Protections (HSP) and Good Clinical Practice (GCP). The trial is monitored by the United States National Institutes for Health (NIH) through Pharmaceutical Project Development (PPD).

### Study status

Not yet recruiting.

### Participants

Participants will be recruited from the general outpatient primary care services at Kisumu County Hospital (KCH). The participants will provide written consent for screening by study personnel, all of whom completed training in Good Clinical Practice and Human Subjects Research through the CITI program.

### Inclusion criteria

- Kisumu County Hospital (KCH) adult primary care outpatient clinic attendees who screen positive for MDD and/or PTSD on the Mini International Neuropsychiatric Interview (MINI)
- Able to attend weekly IPT sessions/fluoxetine monitoring
- 18 years or older.

### Exclusion criteria

Participants will be excluded if any of the following criteria are met:

- Cognitive dysfunction compromising ability to participate in IPT or accurately take fluoxetine (lack of orientation to person, place, time and situation);
- Acute suicidality (moderate or high score on the MINI suicidality module) requiring higher level of care
- Drug/alcohol use disorders requiring substance use treatment (AUDIT score of 8 or higher, DAST score of 3 or higher)
- History of mania or requiring treatment for hypomania (positive score on MINI mania/hypomania module)
- Pregnancy or breastfeeding
- Outside mental health treatment during the study treatment phases (any mental health treatment is allowed during follow-up phases and is recorded by study team). Participants will be asked not to start new mental health treatment during the study treatments, but will be assured that this is not prohibited. Participants who do will be treated as dropouts.

### Interventions

#### Training of non-specialists

##### Interpersonal psychotherapy

Prospective IPT therapists are identified according to the following criteria: 1) fluency in local languages (Dholuo, Kiswahili) and English; 2) plans to remain in the area for the duration of the study; 3) strong interest in providing mental health care; and 4) a promising background for effective communication regarding emotions (e.g., healthcare workers, teachers, church leaders, women’s group leaders, village chiefs/elders, traditional healers, HIV treatment adherence counselors). Prospective IPT therapists join a 5–7-day IPT training course taught onsite by the MPIs, PI and Co-Is with expertise in local delivery of IPT. The course begins with an introduction to mental disorders focused on depression and trauma, emphasizing a medical model. Patient-provider interactions are reviewed, including the fundamental importance of confidentiality. Basic tenets of psychotherapy are introduced, followed by introduction to IPT and instruction on its initial, middle and concluding phases [61]..

In our past work, we adapted IPT for non-specialist, integrated delivery within a FACES HIV care setting. Building on that study, we made minor refinements to optimize logistics and content fit for primary care adult outpatients at KCH. Following the introductory didactics, we began an iterative process of IPT training*e.g., Communication analysis, role playing.

using the local, trainee therapists as our primary resources. We reviewed the sequence of IPT work, including tasks and techniques for each phase and session of IPT. During this review, we solicited feedback from trainees on cultural and logistical adaptations or additions to improve the acceptability and relevance of IPT for the target population. An iterative group process was used to integrate input from trainee therapists for IPT content and process, if consensus was reached that not doing so would negatively affect outcomes or engagement. The adaptations defined through the above processes were incorporated to create an IPT protocol tailored to the needs of KCH adult primary care outpatients.

##### Fluoxetine

Drawing on the experience of our investigative team with training East African non-specialists to deliver fluoxetine [45, 48, 62, 63], we trained interested nurses and/or clinical officers at KCH and other qualified prescribers of the same level living/working in the study area to deliver fluoxetine to study participants.

Prospective fluoxetine providers attended a multi-day training course. As with IPT providers, they began with an introduction to mental disorders, focused on depression and trauma. Patient-provider interactions were reviewed, including the fundamental importance of confidentiality. The use of antidepressants to treat depression and PTSD was taught, focused on the role of fluoxetine. The dosing protocol was introduced, with review of potential side effects and required provider responses for each effect. The importance of ruling out bipolar affective disorder before initiating fluoxetine was emphasized and symptoms/signs of mania/hypomania were reviewed. Role plays and case studies were used to practice assessment, dosing and evaluation for side effects and mania/hypomania.

#### Delivery of study treatments

##### Interpersonal psychotherapy

IPT will be delivered according to standard protocol and will consist of 12 weekly, one hour sessions with the same IPT therapist for each session, conducted in a confidential location at KCH. Participants randomized to receive IPT will meet with the IPT therapist for 12 weekly 60-min sessions.

##### Fluoxetine

Participants randomized to receive fluoxetine will meet with the trained fluoxetine provider in a confidential location in KCH at baseline, 2 weeks, 4 weeks and then monthly until month 6, for a total of eight appointments. Participants will be assessed for initiation of fluoxetine, including evaluation for history of bipolar affective disorder (*see exclusion criteria*) and advisement on common side effects.

Fluoxetine participants will be started on 20 mg 1 tablet per day and will return for assessment of initial efficacy/tolerability at week 2. Barring prohibitive side effects, the dose is continued at 20 mg and the participant returns for re-assessment at week 4. If there is no symptom improvement at week 4 and the medication is tolerated, the dose is further increased to 40 mg (2 tablets). At the 2 month visit, if there is no symptom improvement and the medication is tolerated, the dose will be increased to 60 mg (3 tablets). Studies support that doses of fluoxetine > 60 mg do not shown additional benefit. At the month 6 visit, participants will be given instructions for tapering off fluoxetine, with decrease of the dose by 20 mg increments each week until reaching zero.

#### Competency testing and Fidelity of treatment

##### IPT provider competency testing

At the conclusion of IPT training, prospective study therapists are asked to articulate the fundamental tenets of IPT and to demonstrate an initial, middle, and concluding group IPT session through role play with other trainees. If they demonstrate competency, therapists will be assigned an IPT practice case. If sessions are successfully completed with the training case, as demonstrated by scores of 9 or 10 on our 10–12 item IPT adherence measures administered by local IPT leads and investigators with IPT expertise, the IPT provider will be invited to join the group as a study IPT provider to treat clinical trial participants.

##### Fidelity to IPT protocol

Therapists will discuss each IPT session with the lead IPT therapists through weekly group supervision sessions. Each session will be scored using a 10 point Likert scale assessing adherence in each IPT phase. Sessions will be considered adherent if they average a score of 5 or higher and do not employ off-protocol interventions. IPT lead therapists will be supervised in weekly telephone calls with the investigators, during which time any clinical challenges will be discussed.

##### Fluoxetine provider competency testing

The fluoxetine course concludes with proctored interviews with practice participants, in which prospective fluoxetine providers are asked to apply the fluoxetine treatment protocol and respond to case scenarios including contraindications, side effects and dosing challenges. If they demonstrate competency during these proctored interviews, fluoxetine providers are assigned practice cases. If they successfully complete practice cases, as demonstrated by scores of 9–10 on fluoxetine protocol adherence measures, they are invited to join the study and treat clinical trial participants.

##### Fidelity to fluoxetine treatment protocol

In addition to the measurement of treatment fidelity during the practice cases, fluoxetine providers that join the study are also observed and scored weekly on their adherence to fluoxetine treatment protocol by the study’s lead fluoxetine provider. Local investigators will also randomly select 10 charts of treated patients each month to assess compliance with the fluoxetine treatment protocol (eligibility, dosing and monitoring of side effects). Adherent treatment will have no deviations from protocol.

#### Outcome measures and assessment

All study assessment tools and outcome measures are programmed in REDCap and will be collected offline on tablets, then uploaded daily to a secure server and erased from tablets. All data will be checked daily by the UCSF team for completeness and data quality.

#### Primary outcome measures

- Major Depressive Disorder (MDD) and Posttraumatic Stress Disorder (PTSD) using MINI-MDD and MINI-PTSD modules, respectively, for categorical diagnoses at time points indicated in the table below.
- Beck Depression Index (BDI) and Posttraumatic Stress Checklist (PCL) for continuous measures of depression and PTSD symptom severity, respectively, at the time points indicated in the table below.

#### Secondary outcome measures

- Accessibility*.* We will evaluate accessibility as defined above (geographic proximity to clinic, type and severity of challenge for attending depression appointments)
- Affordability*.* We will assess cost of transportation to KCH and opportunity costs (e.g., lost wages, childcare) for each treatment session
- Domestic violence, alcohol, drugs and trauma. Given the high rates of domestic violence, alcohol use disorders, deaths due to disease such as HIV and the influence of traumatic events on depression etiology and treatment response (e.g., 60) we will measure domestic violence, alcohol and drug use and general trauma history
- Physical health co-morbidities*.* Given the strong association of depression with physical disorders, we will assess for physical co-morbidities
- Health-related disability*.* We will use the WHO measure. All measures have been translated into local languages (Dholuo and Kiswahili) appropriate to the study population’s education level, using a standardized process of measure adaptation and translation [64–68].
- Emotional reactivity. While some assessments of SSRI mechanism of action are not feasible in this setting (e.g., fMRI or PET scans), we are able to assess emotional reactivity, which is thought to be reduced by SSRIs. We will use the Self-Assessment Manikin (SAM) to investigate how emotional reactivity mediates the relationship between treatment sequences involving fluoxetine and remission.
- Social Support. We will use the Multi-Dimensional Scale of Perceived Social Support (MSPSS) to investigate how social processes mediate the relationship between treatment sequences involving IPT and remission.

#### Other variables measured at baseline to characterize the participants

- Age, sex, diagnosis, co-morbidity
- Sociodemographic characteristics (education, job, marital status, individual and household income)

#### Randomization

After completion of baseline measures, participants will be randomized to first-line treatment using a computer generated random sequence applied through REDCap. Randomizations for first and second line treatment will be done in blocks of 12 and stratified to ensure equal size and representation of gender, given the sample size, we expect randomization to achieve approximate balance for other variables.

#### Blinding

Clinical evaluators who conduct follow-up assessments will be blinded to treatment assignment. Participants,IPT/fluoxetine providers and study coordinators will not be blinded.

#### Statistical analysis

For Hypothesis 1) the analysis is modified intention to treat requiring attendance of at least one treatment session. In the post-treatment analyses, we will be comparing the remission at month three for IPT with remission at month six for fluoxetine. We will pool over the second stage interventions to compare the first stage intervention options. Our analysis strategy will be to fit logistic regression models for the outcome of remission (no longer meets MINI criteria for MDD and/or PTSD diagnosis), including as predictors an indicator for the first-stage intervention options, demographics and severity of baseline depression/PTSD symptoms. We expect the two interventions to have similar remission rates at their respective treatment conclusions (3 months and 6 months) and if we are unable to reject the null hypothesis (no significant difference), we will use confidence intervals for the odds of remission to quantify the similarity of the interventions. With our sample size, confidence intervals will be narrow and we will be able to precisely limit differences.

Hypothesis 2) will be analyzed utilizing a logistic regression model to estimate and compare post-treatment relapse for participants who remit with first line IPT to those who remit with first line fluoxetine (ATS groups 1 and 6). The analysis will use generalized estimating equations logistic regression to accommodate the repeated measures over months.

For Hypothesis 3) participants assigned to initial treatment with IPT in the first randomization, will be evaluated for mediation of remission by social support. We will evaluate change in social support (baseline to 1.5 months [m] and 1.5 m to 3 m) for mediation of the relationship between IPT and MDD and/or PTSD remission. Specifically, we will decompose the total causal effect into the natural direct effect and the natural indirect effect, using paramed software [69]. We will calculate the proportion of mediation as the natural indirect effect divided by the total causal effect. For participants assigned to initial treatment with fluoxetine, we will evaluate for mediation of remission by emotional reactivity, using the SAM (Hypothesis [4]). We will assess for mediation using the method described above.

For Hypothesis 5), we will assess how the treatment effect of the first SMART randomization (IPT or fluoxetine) is dependent upon pre-specified moderators of time and cost of transport from participants’ residence to KCH. The primary analysis will be to calculate a 95% confidence interval for the remission rates to quantify the degree of similarity between initial intervention arms. Following this analysis, we will evaluate pre-specified moderators. We will do this by fitting a log-link regression model for the outcome of MDD/PTSD remission post treatment (3 months for IPT or 6 months for fluoxetine) with the predictors of initial group assignment, pre-specified moderator and a moderator by initial group assignment (IPT or fluoxetine) interaction. If the interaction is statistically significant, we will estimate the treatment effect separately for subgroups of the pre-specified moderator. This process will be repeated for time and cost of transport and for both MDD and PTSD remission. Restricting the analysis to non-remitters (those assigned to a second randomization) we will use logistic regression to assess how the effect of the subsequent treatment (switch or combine) is dependent upon pre-specified moderators (severity of depression/PTSD symptoms [BDI/PCL score]) among non-remitters at 3 months (IPT) or 6 months (fluoxetine) (Hypothesis [6]).

We will construct ATS (treatment algorithms) by using identified moderator variables (a) at baseline (e.g., transport time and cost) that predict who will benefit more or less from fluoxetine versus IPT, and (b) at the end of the initial treatment (e.g., severity of symptoms among non-remitters at month 3 [IPT, fluoxetine] or 6 [fluoxetine only]). We will assess for other key demographic (e.g., age, gender) and clinical (social support, emotional reactivity and four health co-morbidities [Table 1]) moderators. This data will be used to identify optimal ATS based on the examined moderators to produce personalized, efficient treatment. To do this, we will apply Q-learning [80] for binary outcome (remission) data by adopting an approach proposed by Moodie and colleagues that uses generalized additive models (Hypothesis [7]) [81]. Q-learning is a generalization of moderated regression analysis to sequences of treatment, used to develop an optimal sequence of decision rules.

**Table 1:** Primary and Secondary Outcome Measures and Data Collection

Variable	Description	Frequency of Assessment
Primary Outcome Measures		
Major Depressive Disorder (MDD)	MINI v. 5.0 MDD module [70]	Prior to enrollment (inclusion criteria), 1.5, 3, 6, 9, 12, 18, 24 and 30 months
Posttraumatic Stress Disorder (PTSD)	MINI v. 5.0 PTSD module	Prior to enrollment (inclusion criteria), 1.5, 3, 6, 9, 12, 18, 24 and 30 months
Secondary Outcome Measures		
Suicidality, mania/hypomania (exclusion-referral)	MINI v. 5.0, suicidality, (hypo) mania modules [70]	Prior to enrollment (exclusion criteria)
Detailed alcohol use (exclusion-referral)	Alcohol Use Disorders Identification Test (AUDIT) [71]	Prior to enrollment (exclusion criteria)
Pregnancy and breast-feeding (exclusion-referral)	Self-report	Prior to enrollment (exclusion criteria)
Drug use (exclusion-referral)	Drug Abuse Screening Test (DAST-10) [72]	Prior to enrollment (exclusion criteria)
Demographics	Age, gender, marital status	Baseline
Highest education	(1) None; (2) primary; (3) secondary; (4) tertiary and beyond	Baseline
Time and cost of transport to KCH	Minutes and KSH for transport between residence and KCH	Baseline
Trauma history	Trauma History Screen (THS) [73]	Baseline
Depression symptoms	Beck Depression Scale (BDI) [74]	Baseline, 1.5, 3, 6, 9, 12, 18, 24 and 30 months
PTSD symptoms	Posttraumatic Stress Checklist (PCL) [75]	Baseline, 1.5, 3, 6, 9, 12, 18, 24 and 30 months
Emotional Reactivity	Self-Assessment Manikin (SAM) [76]	Baseline, 1.5, 3, 6, 9, 12, 18, 24 and 30 months
Domestic violence	Revised conflict tactics scale (CTS2) [77]	Baseline, 1.5, 3, 6, 9, 12, 18, 24 and 30 months
Social Support	Multi-dimensional Scale of Perceived Social Support [78]	Baseline, 1.5, 3, 6, 9, 12, 18, 24 and 30 months
Individual and household income	Assessment of individual and household formal and informal income in the last 30 days	Baseline, 1.5, 3, 6, 9, 12, 18, 24 and 30 months
Health co-morbidities	HIV, Malaria, HTN, DM	Baseline, 1.5, 3, 6, 9, 12, 18, 24 and 30 months
Health-related disability	World Health Organization Disability Assessment Schedule (WHODAS) [79]	Baseline, 1.5, 3, 6, 9, 12, 18, 24 and 30 months
Mood symptoms	PHQ-2	Every IPT and Fluoxetine treatment visit
Risk assessment	Suicidality and homicidality	As needed and if PHQ-2 increases
Adverse events	Any untoward medical occurrence that does not necessarily have a causal relationship with the study intervention	As needed

Diabetes Mellitus (*DM*) Human Immunodeficiency Virus (*HIV*) Hypertension (*HTN*) Kisumu County Hospital (*KCH*) Kenyan Shillings (*KSH*) Major Depressive Disorder (*MDD*) Mini International Neuropsychiatric Interview version 5.0 (*MINI 5.0*) Posttraumatic Stress Disorder (*PTSD*).

We will use data obtained on the economic status of those treated to calculate the economic benefits associated with treated mental health disorders (both pooled analyses of IPT or fluoxetine treatment and separate analyses of IPT and fluoxetine treatment) (Hypothesis [8]). We will also incorporate data on health care utilization and hospitalizations. The value of these economic benefits will be summarized on a per-person basis and then combined with the implementation cost data to generate a rate of return on mental health expenditures (in general and by treatment type –IPT or fluoxetine). This rate of return is net gains in all economic measures divided by implementation costs, × 100 to yield % return. Cost and income measures will be adjusted for inflation and discounting during study period.

#### Data monitoring

The study’s independent, chartered, Data and Safety Monitoring Board (DSMB) reviewed and approved the study protocol for launch. In addition to the United States National Institutes for Health (NIH) Pharmaceutical Project Development (PPD) monitoring (see ethics), the study’s DSMB will review data quality and participant safety annually.

#### Missing data

Prior to analysis, data will be assessed for item-missing data (incomplete data among respondents who participate in most, but not all, of the assessments or assessment components). The problem of item-missing data is endemic to repeated assessment data collection. One of the approaches to address this problem is multiple imputation (MI), which involves generating multiple sets of predicted values of missing data (we will use 10 sets), estimating models separately in each of these replicate datasets, calculated averages across replicates to generate parameter estimates and using information about variation in estimates across replicates to calculate design-based standard errors of parameter estimates. We will create MI replicate datasets using Imputation and Variance Estimation Software, widely-used MI software [82, 83].

#### Sample size estimation

SMART designs are used to inform the construction of optimal ATS, which is our goal. The proposed SMART examines 1) first line treatment strategies with IPT or fluoxetine, including long-term relapse risk for participants who remit with initial treatment and 2) second line treatment options for non-remitters—specifically, treatment “switch” (from IPT to fluoxetine or vice versa) versus treatment “combination” (addition of IPT to fluoxetine or vice versa). We therefore powered our study to detect differences in the smallest branches in the design (see Table 2), namely the second stage randomizations among non-remitters.

**Table 2:** A Sequential Multiple, Assignment Randomized Trial (SMART) of non-specialist-delivered psychotherapy and/or medication for Major Depressive Disorder and Posttraumatic Stress Disorder (DAPPER)

	Enrollment	Allocation	Post-allocation							Close-out
TIMEPOINT		Baseline	*1.5 m*	*3 m*	*6 m*	*9 m*	*12 m*	*18 m*	*24 m*	*30 m*
ENROLLMENT:		X								
Eligibility screen	X									
Informed consent	X									
Random Allocation		X								
INTERVENTIONS:										
*Intervention A*	IPT	X	X	X						
*Intervention B*	F	X	X	X	X					
*Intervention C (non-remitters)*	IPT, IPT (RR)	X	X	X	X					
*Intervention D (non-remitters)*	IPT, F (RR)	X	X	X	X	X				
*Intervention E (non-remitters)*	F, IPT (RR)	X	X	X	X	X				
*Intervention F (non-remitters)*	F, F (RR)	X	X	X	X	X	X			
ASSESSMENTS:										
*Inclusion and Exclusion Criteria*	1. MDD (IC) 2. PTSD (IC) 3. Suicidality (ER) 4. Mania/hypomania (ER) 5. Detailed alcohol use (ER) 6. Pregnancy and breast-feeding (ER) 7. Drug use (ER)									
*Baseline Variables*	1. Demographics 2. Highest education 3. Time and cost of transport 4. Trauma history 5. Depression symptoms 6. PTSD symptoms 7. Emotional Reactivity 8. Domestic violence 9. Social Support 10. Income 11. Health co-morbidities 12. Health-related disability	X								
*Follow-up Variables*	1.MDD 2. PTSD 3. Depression symptoms 4. PTSD symptoms 5. Emotional Reactivity 6. Domestic violence 7. Social Support 8. Income 9. Health co-morbidities 10. Health-related disability		X	X	X	X	X	X	X	X

Exclusion and Referral (*ER*) Fluoxetine (*F*) Inclusion Criteria (*IC*) Interpersonal Psychotherapy (*IPT*) Major Depressive Disorder (*MDD*) Posttraumatic Stress Disorder (*PTSD*) Re-Randomization (*RR*) of non-remitters

As it pertains to antidepressants and psychotherapy, the evidence base comparing “switching” versus “combining” treatment for non-remitters (second line treatment) is still developing [60]. However, the field does have meta-analytic data on the differences between treating depression with antidepressants or psychotherapy alone, versus their combination. A 2009 meta-analysis (*n* = 2036) which compared combination treatment (psychotherapy and pharmacotherapy) to pharmacotherapy alone for depression found an effect size of 0.31 (Cohen’s d) [84]. A 2007 meta-analysis (*n* = 903) found that the remission rate for major depression treated with psychotherapy and pharmacotherapy was better than psychotherapy alone, with an odds ratio of 1.59 [85], which when converted to a Cohen’s d is 0.25 [86]. While specific studies have not yet been completed for IPT and PTSD, we leveraged the above-referenced meta-analyses of the effects of general psychotherapy and general pharmacotherapy to estimate sample size for the proposed study. Based on those studies, we would need approximately 164 per second stage randomization to compare combined psychotherapy-medication treatment with medication alone, and 242 to compare combined psychotherapy-medication with psychotherapy, alone (using a two-sided alpha of 0.05 and power of 0.80). Using the conservative estimate, we will need 242 for each of the four treatment arms (968, total) *after the second randomization* (switch to fluoxetine, switch to IPT, combination after fluoxetine, combination after IPT). While effectiveness data on treatment of depression in HICs (STAR*D) indicate that only approximately one-third remit with first line treatment, preliminary data from our current IPT study in Kisumu indicate remission rates over two-thirds. We will use a conservative estimate of the numbers entering the second randomization phase (non-remitters) and assume that half of study participants will need second-line treatment. Based on that estimate, the 968 participants randomized to the second stage of the SMART should represent 50% of those enrolled at the start of the trial, or 1936. Allowing for a 40% drop out rate across the 33 month duration of the study, we plan to recruit 2710 participants into the SMART. Making a conservative assumption of ~ 20% prevalence of MDD and/or PTSD among primary care patients [87, 88], we will screen 13,552 individuals to enroll 2710 participants. While the clinic sees 10,000 adult primary care patients per month, we will assume that only 20–30% of clinic attendees will undergo screening, and we will budget 6 months, total, for recruitment. In order to avoid overwhelming mental health care providers, these 6 months of recruitment will be dispersed across a 12 month period: enrollment will halt when providers’ caseloads are full and resume when at least 20% availability is regained.

## Discussion

This study will build upon prior work in the field in several impactful ways. First, SMART-DAPPER will use IPT, fluoxetine and combination treatment for mental health care in a LMIC. Despite the fact that structured psychotherapy and SSRIs have equivalent short-term efficacy [89] and are the two most established, evidence-based depression and trauma-disorder treatments in HICs, the vast majority of depression treatment studies in LMICs are efficacy trials of evidence-based psychotherapy—neglecting the potential of medication and combination treatment to alleviate disorders. Only a small number of studies in LMICs examine non-specialist delivery of SSRIs and psychotherapy treatment [90], and, to our knowledge, none have compared their relative treatment outcomes. This study draws on our research team’s experience using non-specialist delivery of IPT and fluoxetine to conduct the first study that includes psychotherapy, SSRI and combination treatment for depression and trauma-disorders in sub-Saharan Africa. By testing evidence-based treatment options heavily used in HICs, this study will provide data essential for advancing global standards of mental health care.

Second, we will use IPT and fluoxetine to address co-morbid MDD and PTSD. While trans-diagnostic treatments are beginning to emerge in global mental health treatment research [91], the majority of studies address single disorders and thus have limited real-world applicability. Given that IPT and fluoxetine are now considered evidence-based treatment for both MDD and PTSD, and our expertise in treating these co-morbidities at FACES, we will deploy IPT and fluoxetine to treat MDD, PTSD or both.

Third, we will integrate mental health treatment with existing primary care. Mental health treatment integrated with primary care is known to have higher efficacy than “stand-alone” mental health care [92], with few exceptions [90]. Yet, the majority of treatment studies in LMICs deliver care in an isolated manner. Our team has conducted formative work on integrated treatment in this region [37, 93, 94]. By testing depression and trauma-disorder treatments delivered within a large, county hospital primary care clinic committed to sustainment (see attached letters), this study will provide data to leverage regional scale-up of integrated mental health care.

In conclusion, this implementation science study of mental health treatment delivery strategies addresses depression and anxiety, leading causes of global disability. Despite carrying the vast majority of the global mental disorder burden, 75% of adults with mental disorders in LMICs have no access to services. This study will use a SMART design to test strategies for local non-specialists to deliver evidence-based mental health care and will inform treatment algorithms essential for personalizing care to achieve rapid remission of mental disorders in low resource settings to efficiently reduce the global mental health burden. Study findings will be disseminated according to the NIH data dissemination policy which is aligned with the ClinicalTrials.gov policy. The ClinicalTrials.gov website will be updated with clinical trial results at least once every 12 months from the date that enrollment begins. Final data results will be uploaded within 12 months of study completion.

## Data Availability

Not applicable.

## Abbreviations

ATS: Adaptive Treatment Strategies
BDI: Beck Depression Index
DAPPER: Depression and Primary Care Partnership for Effectiveness-implementation Research
DAST: Drug Abuse Screening Test
FACES: Family AIDS Care and Education Services
GCP: Good Clinical Practice
HIC: High Income Country
HIV: Human Immunodeficiency Virus
HSP: Human Subjects Protection
IPT: Interpersonal Psychotherapy
KCH: Kisumu County Hospital
KEMRI: Kenya Medical Research Institute
LMIC: Low and Middle-Income Country
MDD: Major Depressive Disorder
MI: Multiple Imputation
MINI: Mini International Neuropsychiatric Interview
MRI: Magnetic Resonance Imaging
MSPSS: Multiple Dimensional Scale of Perceived Social Support
NIH: National Institute of Health
PET: Positron Emission Tomography
PPD: Pharmaceutical Project Development
PTSD: Post-traumatic Stress Disorder
SAM: Self-Assessment Manikin
SMART: Sequential, Multiple Assignment Randomized Trial
SSRIs: Selective Serotonin Reuptake Inhibitors
UCSF: University of California San Francisco
UoN: University of Nairobi
WHO: World Health Organization
